# DOES EXPERIENCE MATTER? A mixed methods study of medical student experiences of near-peer and senior clinician-led tutorials

**DOI:** 10.15694/mep.2019.000148.1

**Published:** 2019-07-08

**Authors:** Katie Bishop, Fiona Rae, Nibu Thomas, Charlie Tombs

**Affiliations:** 1 Undergraduate Department

**Keywords:** near-peer, peers, undergraduate, trainees, tutorials, acute medicine

## Abstract

This article was migrated. The article was marked as recommended.

**Objectives:**The primary aim of the study was to determine whether classroom-based acute care teaching delivered by junior doctors is comparable to that by senior doctors or faculty. This study reviews student opinions of near-peer and faculty led teaching on acute medicine to explore the differences and student preferences.

**Methods:**This study aimed to evaluate the role of trainees as near-peer tutors in the acute medicine tutorial based setting by randomly allocating the sessions to a junior or senior doctor. Student opinions were then invited through questionnaires and focus groups.

**Results:**There was no statistical difference in students’ perception of the level, pace and usefulness of the sessions. All teachers were approachable and enthusiastic. Students felt that senior doctors were more knowledgeable and better able to explain concepts. Students felt that all sessions were useful to their learning.

**Conclusion:**Students enjoyed and derived educational benefit from both types of teaching session. Students found that senior doctor-led tutorials were more beneficial to their technical medical knowledge but felt that they gained more practical advice from junior doctor-led teaching. Trainees could provide reassurance, advice and mentorship regarding their careers and role of a doctor. Students recognised the value of tutorials by junior and senior doctors and requested the inclusion of both in their undergraduate curriculum.

## Introduction

Near-peer education has been defined as teaching by: “a trainee one or more years senior to another trainee on the same level of medical education training” ( Bulte *et al.,* 2007). There is evidence for its efficacy as a teaching resource ( Buckley *et al.,* 2007; Quershi *et al.,* 2013a).

The benefits of near-peer education are multiple. Students describe the personal learning environment created by near-peer tutorials as effective, engaging and stimulating ( Jackson and Evans, 2012). Trainees teaching clinical examination to medical students are thought to be more approachable, organised, trustworthy and passionate than faculty (Quershi *et al.,* 2013b). Near-peers create a ‘safe’ learning environment and are perceived as less threatening than senior doctor ( Ten Cate and Durning, 2007b). A trusting and approachable tutor allows students to feel comfortable in asking questions and subsequently alleviate any concerns or queries (Quershi *et al.,* 2013b)

As recent students, the trainees may be more empathetic to student needs (Millls *et al.,* 2014; Ten Cate and Durning, 2007b). Having recently completed final examinations, near peers provide expert knowledge and understanding of this experience ( Silbert and Lake, 2012; Hill *et al.,* 2010). Simply being further along the career path, tutors become instant role models and provide reassurance to students ( Sarbin, 1976).

Trainees taking on the role of near-peer tutors also benefit from their involvement in medical education ( Lockspeiser *et al.,* 2008; Salerno-Kennedy *et al.,* 2010). In addition to teaching abilities, they also benefit from improvement in clinical skills, self-confidence, communication, and leadership skills ( Salerno-Kennedy *et al.,* 2010; Rashid *et al.,* 2011).

Most medical schools in the United Kingdom have informal near-peer teaching programmes ( Rashid *et al*., 2011). Evidence exists for its use in many settings in both undergraduate and postgraduate environments but this primarily involves bedside or clinical skills lab teaching ( Rashid *et al.,* 2011; Knobe *et al*., 2010; Tang *et al.,* 2014). In this study, we analyse a formal near-peer tutorial programme delivered to 5 ^th^ year Cardiff University Medical School students in a large district general hospital and compare it to tutorials delivered by senior clinicians.

## Methods

Each of the eight 1-hour tutorials in the series were randomised to volunteer F1 or senior doctor tutors. All students studying on the Junior Student Assistantship module in Year 5 were invited to attend each of the tutorials:

1. Airway
2. Breathing
3. Circulation
4. Disability
5. Exposure
6. Identifying the unwell patient (including NEWS) and communicating with other healthcare professionals (including SBAR)
7. Medical Emergencies
8. Surgical Emergencies

There were two components to the study:

1. Questionnaires following each session
2. Focus groups following the teaching programme

(See Supplementary File 1)

Content analysis of individual questions and focus group transcripts took place to identify categories and common criteria. All data was analysed in Microsoft Excel is to determine totals, percentages and averages. These were then compared and P-values calculated to determine significance of results.

Ethical approval was obtained from the Bangor University Research Ethics Committee.

## Results/Analysis

All 14 students in the module took part in the programme. Attendance rate was 89.29% overall with no significant difference in the attendance rate between the junior and senior doctor led sessions (p=0.05).

**Figure 1.**
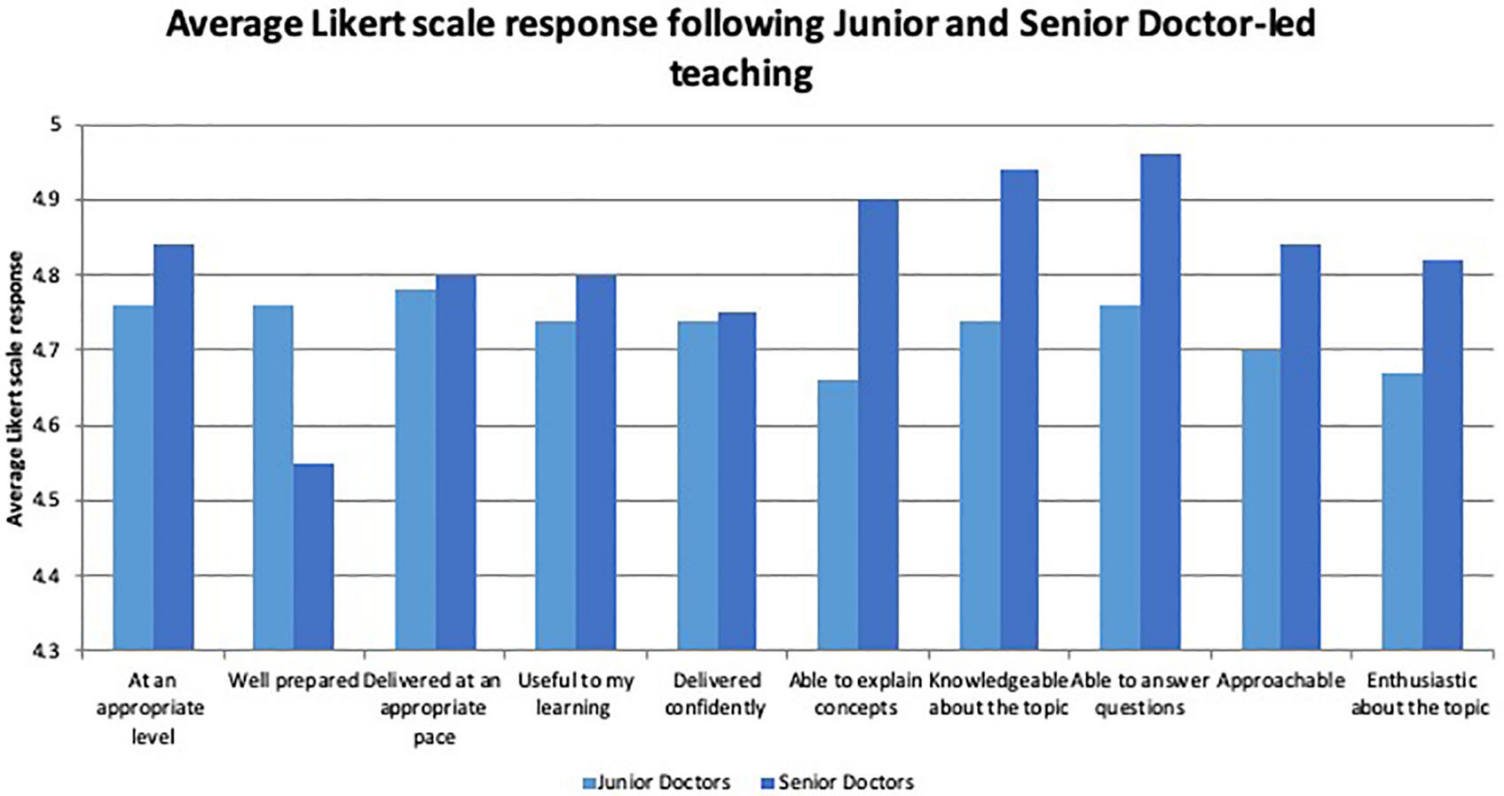
Comparison of mean response form Likert Scale responses in both Junior and Senior Doctor Groups

**Questionnaire responses** Students felt that all teaching sessions were delivered at the appropriate level and pace. All doctors were prepared to teach and created confidently delivered tutorials that were useful to learners. There was no statistical difference in the mean Likert scores between the two groups in any of these domains ( Figure 1).

Students felt that both groups had sufficient experience to teach but that senior doctors were more knowledgeable and therefore better able to explain concepts and answer questions. Both groups were approachable and enthusiastic with no statistical difference in the mean Likert score in each group ( Table 1).

**Table 1.** Comparison of results from Likert Scale Responses in both Junior and Senior Doctor Groups

		Junior Doctors	Upper	Lower	Median	Mode	Senior Doctors	Upper	Lower	Median	Mode	P-value	F-value	dF	T-value
**The teaching session was**	At an appropriate level	4.76±0.42	4.88	4.64	5	5	4.84±0.36	4.94	4.75	5	5	0.22	1.40	103	1.22
	Well prepared	4.76±0.52	4.90	4.62	5	5	4.55±0.83	4.77	4.33	5	5	0.18	2.56	91	1.33
	Delivered at an appropriate pace	4.78±0.46	4.91	4.65	5	5	4.80±0.39	4.91	4.70	5	5	0.65	1.43	96	0.45
	Useful to my learning	4.74±0.44	4.86	4.62	5	5	4.80±0.51	4.94	4.67	5	5	0.20	0.75	103	1.98
	Delivered confidently	4.74±0.48	4.87	4.61	5	5	4.75±0.54	4.89	4.60	5	5	0.41	0.80	10.	1.98
**The teacher was**	Able to explain concepts	4.66±0.48	4.79	4.53	5	5	4.90±0.29	4.98	4.83	5	5	0.002	2.72	79	3.19
	Knowledgeable about the topic	4.74±0.44	4.86	4.62	5	5	4.94±0.23	5.00	4.88	5	5	0.0004	3.74	72	1.99
	Able to answer questions	4.76±0.43	4.88	4.64	5	5	4.96±0.19	5.00	4.91	5	5	0.003	5.21	66	3.08
	Approachable	4.70±0.61	4.87	4.53	5	5	4.84±0.36	4.94	4.75	5	5	0.12	2.98	77	1.56
	Enthusiastic about the topic	4.67±0.47	4.80	4.54	5	5	4.82±0.37	4.92	4.72	5	5	0.06	1.59	93	1.87

In free text responses, students commented that they gained more medical knowledge from senior doctors but more practical advice from F1s ( Table 2). Respondents preferred the practical advice and approachability of the F1s. They felt that senior doctor-led tutorials could benefit from more lesson planning but enjoyed their interactivity.

**Table 2.** Response to Free Text Questions

Question	Response	Senior Doctor-led tutorials	F1-led tutorials	Z-score	P-value
What did you learn from this session	Medical knowledge	83.64% (n=46)	58.00% (n=29)	2.9	0.004
	Practical advice	0.00% (n=0)	30.00% (n=15)	4.39	0
	No response	16.36% (n=9)	10.91% (n=6)		
What did you like most about this session?	Medical knowledge	9.10% (n=5)	8.00% (n=4)	0.19	0.84
	Practical advice	5.45%(n=3)	34.00% (n=17)	3.75	0.0002
	Approachable tutor	0.00% (n=0)	12.00% (n=6)	2.65	0.008
	Interactive session	30.91% (n=17)	14.0% (n=7)	2.06	0.04
	No response	54.55% (n=30)	29.09% (n=16)	3.53	0.0004
How could this session be improved	Improved/further medical knowledge	0.00% (n=0)	4.00% (n=2)	1.5	0.13
	More Practical advice	0.00% (n=0)	4.00% (n=2)	1.5	0.13
	Increased lesson planning	14.55% (n=8)	0.00% (n=0)	2.81	0.005
	No response	85.45% (n=47)	84.00% (n=46)	1.05	0.29

**Focus group responses** Participants were complementary of both the junior and senior doctors, agreeing that the programme was beneficial to their learning. They commented on the informal mentorship created throughout the teaching programme. Students understood understand what is expected of them when they qualify and benefitted from the insight of current F1s in order to learn their trade. This was summarised by one student who commented:

“They know what we want to know and hear”

Participants agreed that becoming a doctor is one of the most daunting experiences in their lives. Many did not have a vast amount of practical experience of the job and were concerned about their ability to cope. One tutorial, although not directly aimed at this, became a forum for general concerns and questions about becoming a doctor.

When asked about the benefits of F1-led teaching, participants stated that the informal nature of teaching by F1s was beneficial to their learning. This aided discussion and allowed students to ask questions without fear of ridicule or embarrassment. One added:

“ *we could ask the stupid questions that you can’t ask anyone else*”

In general, participants felt that both junior and senior-doctor led teaching was beneficial. There was a general consensus that both should be included in the curriculum. One participant summarized the groups’ responses:

“It doesn’t matter as much who is teaching us.. An F1 will give you an F1 perspective which is valuable.. Whereas a Consultant will give you their perspective which is also valuable.. I think you learn different things.. and you need them both”

They commented that it was not possible to choose a preferred group (junior or senior teachers). In contrast, the best tutors were not from either group but those with enthusiasm for teaching and willingness to share their knowledge.

## Discussion

The main finding of this study is that medical students benefit from both senior and F1-led teaching. They subsequently advocated taking part in the teaching programme. Tutorials delivered by trainees and senior doctors were both at the appropriate level and pace for students’ learning. This illustrates that teachers from both groups have adequate knowledge of the curriculum and what is expected of students at each stage in their training. This is in contrast to other studies which suggest that trainees are more aware of student needs as a result of the limited cognitive congruence between junior doctor and senior medical student ( Bulte *et al*., 2007). Previous studies have shown that trainees are approachable (Quershi *et al.*, 2013b). This study adds that senior doctor tutors are also approachable to students, leading to development of rapport between senior doctor and medical student. This finding could be related to the self-selected group of doctors who are actively involved in medical education and therefore its external validity is uncertain. Senior and F1 teachers in this programme were both confident and enthusiastic. However, questionnaire responses demonstrated that senior doctor teachers were more knowledgeable about their topic. Consequently, this made them better able to explain concepts and answer questions than F1s. Despite this, F1s possess sufficient knowledge to teach medical students. Participants recognised that although F1s were not experts in their field, they were relative experts. This is because despite having less clinical experience than their seniors, F1s are considerably more knowledgeable when compared to their students. As such, near peers assume the role of expert alongside adoption of the teacher role ( Heckmann *et al.,* 2008). Level of knowledge could affect the preparation work completed by tutors. Students recognised that senior and junior doctors were both prepared to teach but felt that F1s were more primed than their senior colleagues. This could be a result of F1s’ insight into their lack of expertise with compensatory study and preparation to avoid appearing unwise in front of students ( Bargh and Schul, 1980). Participants recognised that the value of near-peer teaching extends beyond the acquisition of clinical knowledge. Through spending time with role models, students were able to develop aspects of the hidden curriculum ( Karnieli- Miller *et al.,* 2010; Nuetens, 2008). A student’s exposure to trainees provides the opportunity to discuss clinical and non-clinical aspects of graduating and becoming a doctor. Many stayed behind after tutorials to question F1s further about their experiences. Through these discussions, students concerns were addressed and they developed an understanding of what is expected when they qualify. They were reassured and gained confidence in their ability to practice medicine, thus assisting the transition from student to doctor.

## Limitations

This study included a small number of participants. In view of this, the crossover method was utilised in study design. Therefore, the ‘carry over’ effect described in therapeutics trials could apply in this context. Additionally, we did not match F1 and senior doctors by their training in medical education as this data was not collected. Questionnaires were completed in the presence of their teacher which may affect the truthfulness of responses. Similarly, focus groups were conducted by the researcher and although not involved in the teaching of this programme, may be known to the participants from previous placements.We were not able to determine the effect of the teaching programme on assessment outcomes or effect on starting work. Further research will involve extending the study to other modules and years to determine the generalisability and transferability of its conclusions to other areas of medical education.

## Conclusion

Teaching delivered by F1s is comparable to that delivered by senior doctors. Students valued teaching sessions from both senior and F1s, finding all relevant to their learning. The knowledge from each tutorial gained is different depending on the tutor’s level of clinical experience. Students prefer near-peers to provide practical advice for taking on the role of a doctor and provision of mentorship. However, they chose to be taught technical information including scientific basis of disease from senior clinicians. Students recognised the importance of a combination of these elements in their undergraduate training and requested that their curriculum contains a combination of tutorials with trainees and senior doctors. This study adds to current knowledge regarding near-peer education. Previous studies have proven the effectiveness of near-peer teaching in clinical skills and bedside teaching. This study adds evidence for its effectiveness in formal tutorial based settings.

## Take Home Messages

• Teaching delivered by F1s is comparable to that delivered by senior doctors.
• Students valued teaching sessions from both senior and F1s, finding all relevant to their learning.
• Students requested that their curriculum contains a combination of tutorials with trainees and senior doctors.
• Trainees are a valuable and useful teaching resource.

## Notes On Contributors

Katie Bishop- Teaching Fellow, SAS Doctor in Geriatric Medicine, North Wales Clinical School, Wrexham, Betsi Cadwaladr University Health Board.

Fiona Rae- Honorary Senior Lecturer, Cardiff University, Consultant in Emergency Medicine, North Wales Clinical School, Wrexham, Betsi Cadwaladr University Health Board.

Nibu Thomas- ST5 Geriatric Medicine, North Wales Clinical School, Wrexham, Betsi Cadwaladr University Health Board.

Charlie Tombs-Teaching Fellow, LAS CT1, North Wales Clinical School, Wrexham, Betsi Cadwaladr University Health Board.
