# Supplementary material for: DOES EXPERIENCE MATTER? A mixed methods study of medical student experiences of near-peer and senior clinician-led tutorials
Source: MedEdPublish (2016). 2019 Jul 8;8:148. [Version 1] doi: 10.15694/mep.2019.000148.1 (PMC10712465; doi:10.15694/mep.2019.000148.1)
Supplement: Peer review history

# Reviewer report: Reviewer response for version 1

**DOI:** 10.21956/mep.19867.r30020

**Published:** 2019-10-04

**Authors:** Elisabeth Schlegel

**Affiliations:** 1 Donald and Barbara Zucker School of Medicine at Hofstra/Northwell

This review has been migrated. The reviewer awarded 4 stars out of 5 This is an interesting study confirming the importance of near-peer education in medical education. As many medical education environments, the sample size was small and the authors leveraged both qualitative and quantitative methods. My only concern is the mentioning of the crossover method in the limitations. This concept requires an introduction and explanation of the application in the design of this study.

Reviewer Expertise:

NA

No decision status is available

# Reviewer report: Reviewer response for version 1

**DOI:** 10.21956/mep.19867.r30019

**Published:** 2019-08-01

**Authors:** Irine Sakhelashvili

**Affiliations:** 1 European University

This review has been migrated. The reviewer awarded 5 stars out of 5 Very interesting and very valuable study. Considering that the modern teaching processes is/ should be a student-oriented process, is necessary to know what the attitude of a student's toward the academic personnel... What do they prefer? Why? Particularly important is to understand the factors linked to the academic achievement. Quality of education depends on the many factors. At the first glance, the age of tutors seems not to be relevant to this issue but as we know already and as this study shows the teacher's age also matters. The aged professionals are better for their great experience, for their fundamental knowledge, but young tutors also have a big advantages: enthusiasm, motivation, they are speaking in the same language as the students are speaking….lot of pluses; So, senior and young tutors might have a positive impact on the students achievement with different manner; obviously both are and should be respected contributors to the teaching process. This opinion was already confirmed by present study. Only one note: there is very small size of the participants. It would be very interesting to see a results from further study with the larger sample size.

Reviewer Expertise:

NA

No decision status is available

# Reviewer report: Reviewer response for version 1

**DOI:** 10.21956/mep.19867.r30018

**Published:** 2019-07-14

**Authors:** Balakrishnan Nair

**Affiliations:** 1 Centre for Medical Professional Development and University of Newcastle

This review has been migrated. The reviewer awarded 4 stars out of 5 This is an interesting studyNear to peer teaching is important and has benefits for the junior student and senior trainee; both groups learn from this experience. For example , I can explain the concept and but the senior trainee had the most recent lived in experience and can give the student more hands on advice (for example how to do an IV cannulation )It would have been better to explain the Cross over method in the Methods section Being of a small sample size and single centre study, it may not be generalisable. But there are many lessons from this paper

Reviewer Expertise:

NA

No decision status is available

# Reviewer report: Reviewer response for version 1

**DOI:** 10.21956/mep.19867.r30021

**Published:** 2019-07-10

**Authors:** Felix Silwimba

**Affiliations:** 1 University of Lusaka

This review has been migrated. The reviewer awarded 5 stars out of 5 this is good study and relevant to medical education. it confirms continuity in medical education . I recommend it to all medical educators. to pick lessons from it. that no medical practitioner should be left behind in medical education.

Reviewer Expertise:

NA

No decision status is available
